# Comment on Chao, H.-C. Zinc Deficiency and Therapeutic Value of Zinc Supplementation in Pediatric Gastrointestinal Diseases. *Nutrients* 2023, *15*, 4093

**DOI:** 10.3390/nu16010134

**Published:** 2023-12-30

**Authors:** Anil K. Verma, Shilpa Tandon, Jedid-Jah Blom, David Armstrong, Maria Ines Pinto-Sanchez

**Affiliations:** 1 Department of Medicine, Farncombe Family Digestive Health Research Institute, McMaster University, Hamilton, ON L8S4K1, Canada; vermaa61@mcmaster.ca (A.K.V.); tandos1@mcmaster.ca (S.T.); armstro@mcmaster.ca (D.A.); 2 Digestive Diseases Clinic, Hamilton Health Sciences, Hamilton, ON L8S4K1, Canada; blom@hhsc.ca

We appreciate the recent review article by Chao H.-C. [1]: “Zinc Deficiency and Therapeutic Value of Zinc Supplementation in Pediatric Gastrointestinal Diseases.” The author has highlighted an important topic as zinc deficiency (ZD) affects up to 64% of untreated [2,3,4] and up to 40% of CeD patients already following a strict gluten-free diet (GFD) [4].

The review focused on the pediatric population, reporting that untreated pediatric CeD patients exhibit significantly reduced serum zinc levels which subsequently returned to normal upon adopting a GFD [5,6]. Zinc deficiency is also highly prevalent in adult CeD patients before diagnosis and may persist despite a GFD [2,3,7]. Furthermore, patients without CeD who adopt a GFD have a similarly increased risk of ZD, suggesting that this may be related to dietary composition rather than malabsorption [7]. Zinc is the second most abundant micronutrient in the human body [8]. It is well-known that ZD impacts health negatively, leading to delayed sexual development in adolescents, increased risk of infections and decreased wound healing, with other complications including diarrhea and neurological changes such as cerebellar ataxia and dementia [9,10]. The main strategies for combating ZD include dietary modification and supplementation [9]. A specific diet may be a sustainable option to treat micronutrient deficiencies [9]; however, as mentioned [1], there are no clear guidelines on how to provide dietary zinc supplementation, and research on the effect of a zinc-rich diet is lacking. Oral supplements are the first choice to treat ZD; however, they have been associated with adverse events including digestive issues which impact treatment compliance.

Therefore, there is a strong need for studies to evaluate the most effective and best-tolerated zinc supplementation not only in pediatric patients but also in adult CeD patients. Since dietary zinc intake may vary depending on dietary pattern, studies from different countries are needed to identify zinc status and the most appropriate treatment in populations adopting different diets. Collaboration is needed to raise awareness of ZD in CeD as restoring zinc levels will likely result in decreased complications and improvement in overall health in populations adopting a GFD.
